# Pregnancy‐Related Aortic Dissection—A Rare Complication of Pregnancy

**DOI:** 10.1002/ccr3.70090

**Published:** 2025-01-06

**Authors:** Mohammad Baseem Shaikh, Reagan Michelle Stafford, Erinn Ogburn, Samuel Tyagi

**Affiliations:** ^1^ Division of Hospital Medicine University of Kentucky Lexington Kentucky USA; ^2^ Department of General Surgery University of Kentucky Lexington Kentucky USA; ^3^ Division of Cardiothoracic Surgery University of Kentucky Lexington Kentucky USA; ^4^ Division of Vascular and Endovascular Surgery University of Kentucky Lexington Kentucky USA

**Keywords:** endovascular repair, malperfusion, pregnancy‐related aortic dissection, type A aortic dissection

## Abstract

Patients with pregnancy‐related aortic dissections should be transferred to high‐volume aortic centers for management. Malperfusion syndromes from acute type A aortic dissections appear to have better outcomes with thoracic endovascular repair before definitive surgical repair.

## 1 Introduction

Pregnancy‐related aortic dissections are rare, fatal complications of pregnancy, occurring in about 0.0004% of pregnancies [1]. They account for 0.1%–0.4% of all aortic dissections. Pregnancy‐related aortic dissections account for 19% of aortic dissections occurring in women less than 35 years of age [2]. Women at highest risk of dissection are those with underlying aortopathies such as Marfan syndrome, Loeys–Dietz syndrome, and vascular Ehlers–Danlos Syndrome, and Turner syndrome, in addition to nonsyndrome aortopathies including nonsyndromic heritable thoracic aortic disease (HTAD) and bicuspid aortic valve with aneurysm [3]. Nonsyndromic HTAD due to pathogenic genetic variants accounts for many cases of aortic aneurysm and dissections. Many women do not exhibit outward features of aortopathies and thus are not identified until after a dissection has occurred [4]. Historically, the DeBakey classification of aortic dissections divided them into three types based on the origin of the intimal tear and extent of dissection: Type I, with tear originating in the ascending aorta and involving distally the ascending aorta and descending aorta; Type II, with dissection tear limited to the ascending aorta; and Type III, with dissection tears originating in the descending aorta [5]. Simpler to use, the Stanford classification divided dissections into Type A, with involvement of the ascending aorta and its main branches, and Type B, without branch involvement, with dissection involving only the descending aorta [6]. Pregnancy‐related aortic dissections are most commonly type A acute aortic dissection (60%–90%), with most occurring during the third trimester and within 12 weeks postpartum [7]. More recently, the Society of Vascular Surgery and the Society of Thoracic Surgery [8] suggested using a novel classification system that defines the aortic dissection anatomy according to the location of intimal tears and the proximal and distal extent of the dissection process using aortic landing zones [8]. In the era of endovascular stent‐grafting, clinical success is often determined by the proximal sealing zone.

Acute aortic dissections occur due to the formation of an intimal tear, allowing blood to pass into a false lumen surrounded by the aortic intima and aortic media. The dissection flap has the capability of propagating proximally or distally. The dynamic relationship of the true lumen and false lumen influences the perfusion of distal organs. The tear leads to loss of transluminal pressure in the true lumen and thereby collapses the walls while simultaneously the false lumen fills and expands with blood, further collapsing it [6]. Malperfusion can occur through various mechanisms, such as when the dissection tear extends into the branches and causes mechanical compression or thrombosis, or if the dissection flap prolapses into the ostia of the vessels. Malperfusion syndrome is the clinical evidence of end‐organ damage as a consequence of malperfusion. Pregnancy results in hormonal changes and physiological increases in stroke volume, cardiac output, and end‐diastolic dimensions, impacting the shear stress the aorta encounters. The peak of hemodynamic stress occurs during the third trimester and postpartum period, accounting for the timing of the dissection.

Although the classical presentation of abrupt onset, severe, sharp chest pain radiating to the back is most often reported, patients can have various presentations based on the extent of the dissection [4]. Nonspecific symptoms of pregnancy can mimic aortic dissection such as back pain, dyspnea, syncope, or weakness. Additionally, providers would be hesitant to order diagnostic computed tomography (CT) because of concern for fetal harm. The radiation exposure with imaging modalities such as CT is lower than the dose needed for fetal harm, especially by third trimester [9]. This often leads to delays in diagnosis and complications ensue. However, the CT with contrast is indispensable in the management of aortic dissection because of rapid acquisition, sensitivity, specificity, the extent of the tear, and evidence of malperfusion or malperfusion syndrome. Bedsides, transthoracic echocardiography can also be useful in the evaluation of patients to detect complications such as aortic valve regurgitation, left ventricular dysfunction, and cardiac tamponade. MRI imaging can also be utilized but is often limited by center availability, image acquisition times, and patient hemodynamic instability.

Patients with aortic dissections can also present with symptoms that are consequences of malperfusion syndromes. Cardiac complications include aortic regurgitation, myocardial ischemia or infarction, cardiac tamponade, or cardiogenic shock. Neurological symptoms such as altered consciousness, focal deficits, stroke, syncope, and paraplegia occur with malperfusion of involved arch vessels. Lower extremity pain and ischemia can occur with occlusion of or dissection extension into common iliac arteries. Mesenteric ischemia may also occur if mesenteric vessels are involved and cause bowel ischemic or necrosis. We present the case of a pregnancy‐related aortic dissection complicated by malperfusion syndrome who presented 3 weeks postpartum and managed at our institution.

## 2 Case History and Examination

36‐year‐old G6P3 + 3 female with medical history of substance use disorder in remission, ectopic pregnancy, spontaneous abortion presented to the emergency room with altered mental status. There was concern she had return‐to‐use prior to her presentation. Three weeks ago, she had an uncomplicated induced vaginal delivery of a healthy term baby girl.

Upon presentation, she was agitated and combative with care with vital signs of temperature of 35.7 (96.3 F), heart rate 92, respiratory rate 25, BP 147/93, and oxygen saturation of 99% on room air. Her Glasgow Coma Scale score was 12/15 (E3 M5 V4). She had audible S1 and S2, with no detected murmurs. Her lungs were clear and abdomen was soft and nontender. She had no limb swelling and had palpable dorsal pedis pulses. Her initial complete blood count was normal. Her electrolytes revealed bicarb of 13 mmol/L, anion gap of 23 mmol/L, and lactate 5.6 mmol/L. Her creatine kinase (CK) was elevated to 11,312 U/L. Her urine drug screen was positive for methamphetamines. Her chest X‐ray demonstrated bilateral interstitial airspace disease.

16 h after her presentation, she began complaining of left leg pain, and her abdomen was mottled. On repeat examination, her abdomen had a reticular rash, and she had diminished pretibial and dorsal pedis pulses in her left leg. She had no discoloration, pallor, or wounds on her extremities.

**FIGURE 1:**
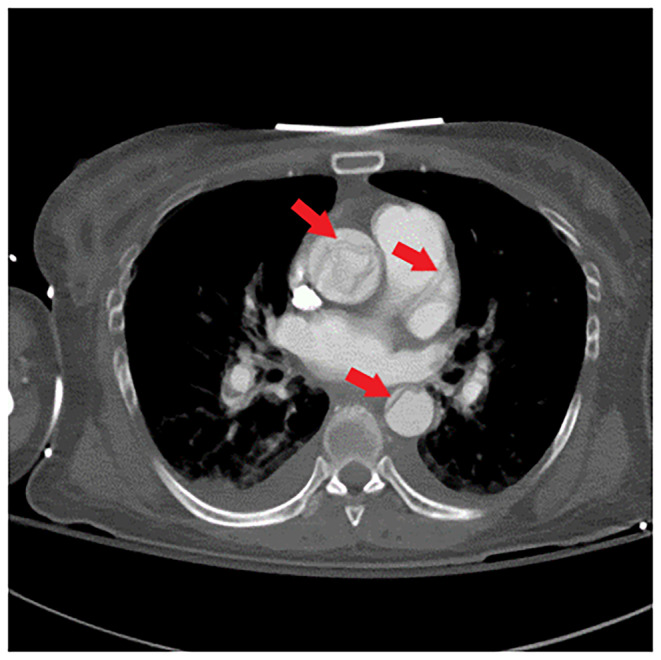
Axial image of CTA chest demonstrates type A dissection flap involving the thoracic aorta (red arrows), which extends inferiorly to the level of the aortic valve. Dissection flaps extend into the proximal portions of all three arch vessels, with more superior extension into the left common carotid artery.

**FIGURE 2:**
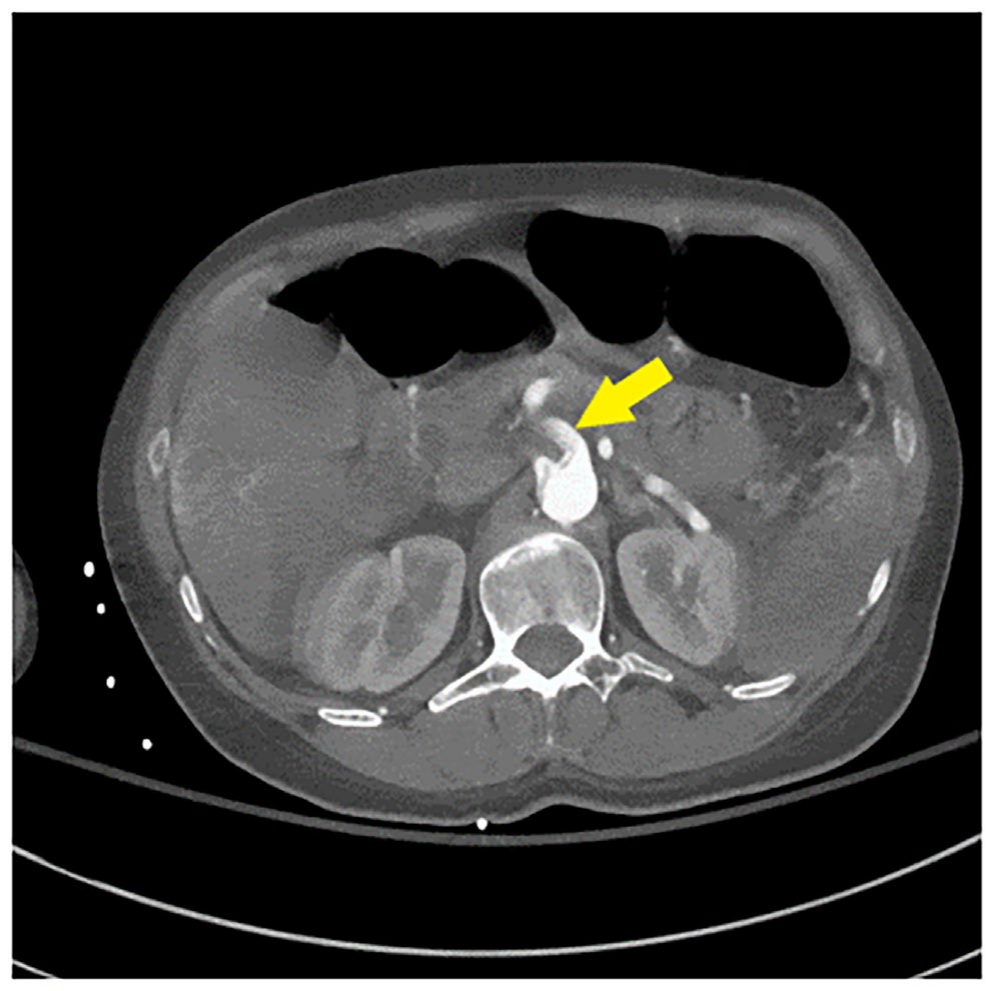
Axial images of CTA of abdomen demonstrates extension of dissection flap extending into the promixal superior mesenteric artery. The celiac, left renal and inferior mesenteric artery all arise from the false lumen. (yellow arrow). Right renal artery arises from the true lumen.

**FIGURE 3:**
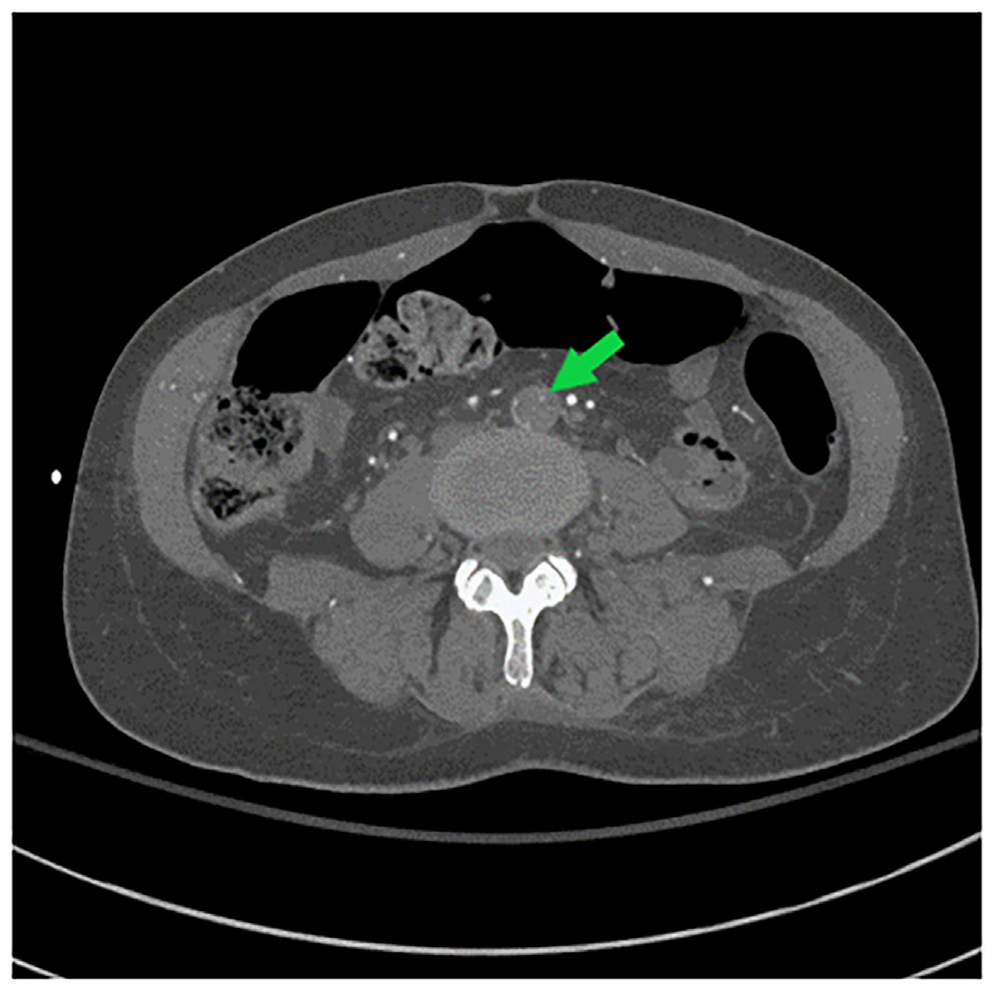
Axial image demonstrating occlusion of infrarenal aorta (green arrow), with extension of the dissection into both common iliac vessels. There is near occlusion of the left common and left external iliac artery. The right common and right external iliac arteries are moderately stenotic.

Her repeat CK was up trended to 217,050 U/L. CT angiogram of the head, neck, chest, abdomen, and pelvis (Figures 1, 2, 3) demonstrated an intimal tear arising in zone 0 of the ascending aorta. The dissection flap extended into the left common carotid artery to the level of the carotid bifurcation. The dissection flap extended into only the proximal portion of the left subclavian and brachiocephalic arteries, without evidence of obstruction. There was no evidence of the dissection involving the vertebral arteries. Right and left main coronary arteries arise from the true lumen and appear to perfuse normally. Distally, the dissection flap extends into the proximal SMA. The celiac axis, left renal artery, and inferior mesenteric artery all arose from the false lumen. The distal aorta appeared to be completely occluded, with the dissection propagating further to involve both common iliac vessels. The left common and left external iliac arteries had near‐complete occlusion. The right common and external iliac arteries were moderately stenotic. The common femoral, superficial, and profund femoral arteries were widely patent bilaterally. However, more distally the left popliteal artery, anterior tibial artery, posterior tibial, and peroneal arteries were occluded. On the right, anterior tibial and peroneal artery were patent to the level of the ankle; the right posterior tibial artery was patent throughout and supplied the plantar arch and right foot, including the descending to the bilateral iliac arteries and more proximally into the arch vessels. There was aortoiliac occlusions with the dissection extending into the superior mesenteric artery. The celiac, inferior mesenteric and left renal arteries arose from the false lumen.

## 3 Management

Given the patient had malperfusion syndrome of abdominal aortic branches and occlusion of the distal aorta and left common iliac artery, with developing compartment syndrome, the plan was to first correct her malperfusion and allow her time to recover, followed by open root repair. She was started on a continuous infusion of esmolol to target a blood pressure of < 120. She was taken to the operating room (OR) and her bilateral groins and abdomen were prepped and draped in a sterile fashion. Bilateral common femoral arteries, superficial femoral arteries, and deep femoral arteries were evaluated with ultrasound to confirm patency, using micropuncture needles followed by advancement of microwires into the infrarenal aorta. Microwires were exchanged for microcatheters. Glide wires were then advanced to the ascending thoracic aorta. After confirming placement of the wire in the true lumen, a 37 × 200 mm Gore Conformable Thoracic Aorta Stent Graft (CTAG) was advanced, with the proximal edge distal to the left subclavian artery (zone 2). An additional 37 × 100 mm CTAG was advanced to extend the coverage to the distal edge, proximal to the celiac artery. A COOK TX2 dissection stent was deployed in the abdominal aorta to treat the dissection down to the terminal aorta. Complete angiogram from the aortic valve to the external iliac arteries revealed complete exclusion of the false lumen and re‐expansion of the true lumen.

For her compartment syndrome, she underwent bilateral lower leg fasciotomies of the posterior and anterolateral compartments; the muscle compartments that were released had moderate bulging present.

Following thoracic endovascular repair (TEVAR), she returned to the intensive care unit (ICU) for postoperative monitoring to ensure resolution of malperfusion. She was started on naloxone infusion for 48 h for spinal cord protection. On Day 2, she was optimized to return to the OR for aortic repair. She was found to have complete circumferential dissection of the intima with extension to the coronary ostia and valve annulus. Intraoperative transesophageal echo showed no evidence of aortic valvular or perivalvular leak and had good flow through both coronary ostia. She underwent aortic root replacement, with 23‐mm freestyle graft, ascending aortic replacement with 24‐mm Hemashield graft and aortic hemiarch replacement with 24‐mm Hemashield graft.

## 4 Outcomes

**FIGURE 4:**
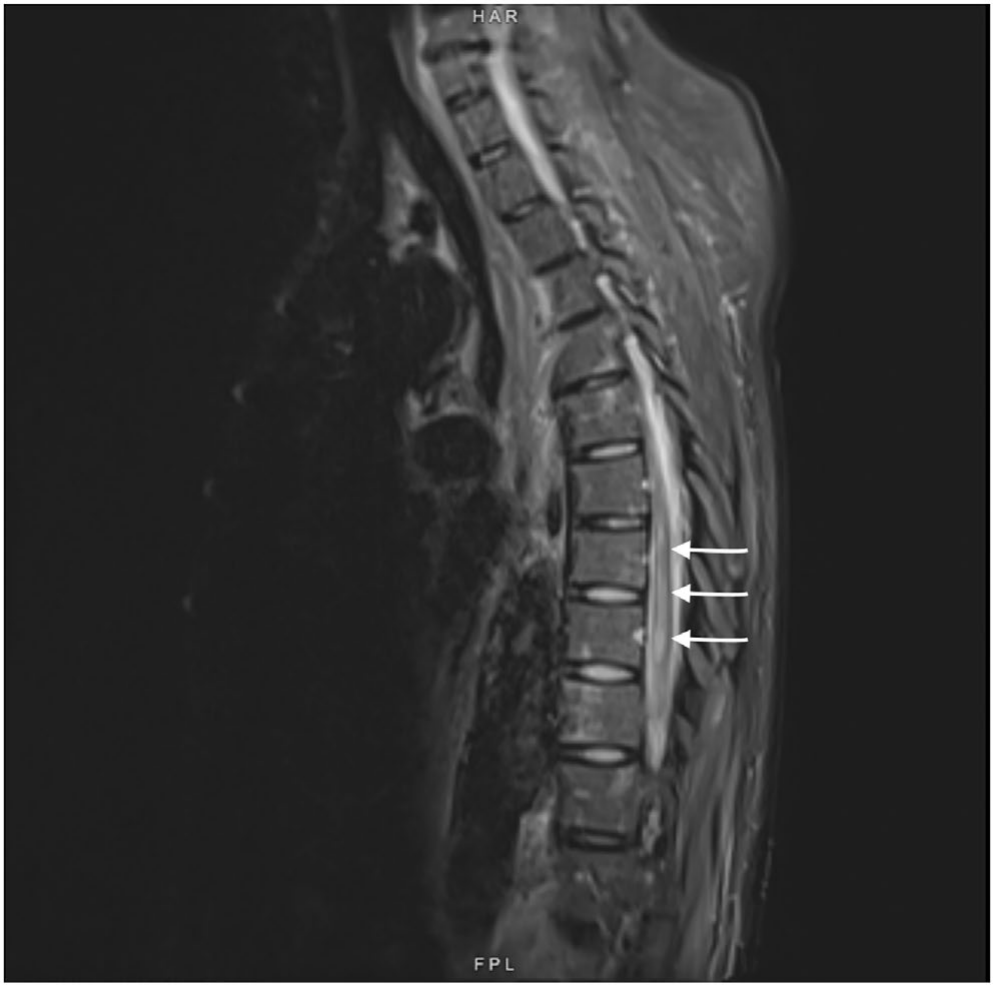
MRI T2 sagittal view of thoracic spinal cord. There is abnormal T2 hyperintensities (white arrows) in T7‐T9 spinal cord suggestive of cord infarction.

**FIGURE 5:**
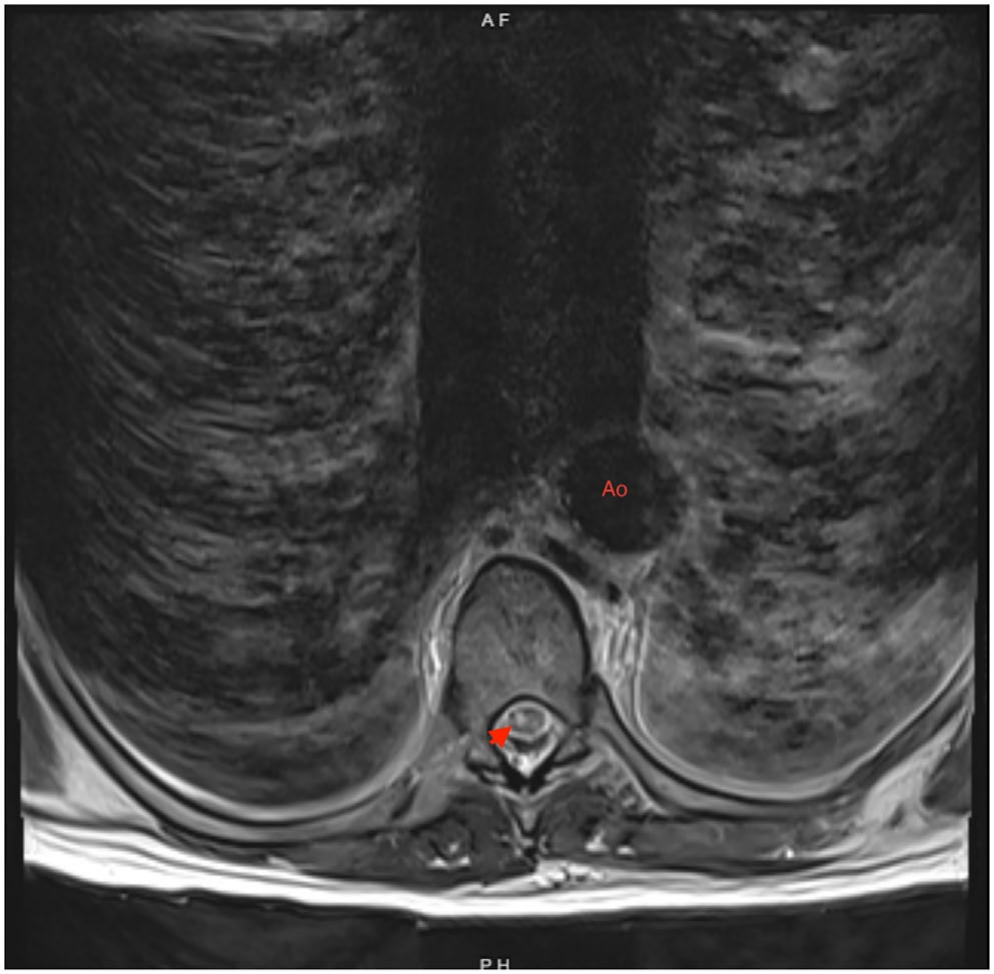
MRI T2 axial view of thoracic spinal cord. There is abnormal T2 hyperintensity (red arrow) within the spinal cord suggestive of cord infarction. Note the aorta with patent endovascular graft (Ao).

On postoperative Day 4, she complained of reduced sensation and weakness in her bilateral lower extremities. She was started on aspirin out of concern for ischemic strokes. An MRI obtained 3 days later of her brain showed multiple scattered acute infarcts in varying vascular territories with embolic phenomenon. MRI spine (Figures 4 and 5) showed nonspecific abnormal signal within distal thoracic spinal cord suggestive of cord infarcts. At 3 weeks post op, she was discharged to rehabilitation. At her 1 year follow up, she remained paraplegic and had developed stage IV sacral pressure ulcer.

## 5 Discussion

Given the complexity and rarity of the condition, conclusive recommendations are limited and usually dictated by local expertise, with extrapolation of data from nonpregnancy‐related aortic dissections. Patients with pregnancy‐related acute type A aortic dissection should be transferred to a high‐volume aortic center to improve survival [6, 9]. Management of pregnancy‐related acute aortic dissection is governed by the type (A vs. B) and the gestational age [3]. In patients with type A aortic dissection and gestational age < 28 weeks, maternal survival should be prioritized given the elevated risk of fetal death. Urgent surgical repair is recommended, with aggressive fetal monitoring or abortion, depending on the extent of the dissection and discussion with the patient and family members. After 28 weeks, single‐stage cesarean delivery and aortic repair is preferred. In patients with uncomplicated type B aortic dissections, medical management, including impulse control with aggressive maternal and fetal monitoring, is warranted. If complicated after 28 weeks, like type A dissections, single‐stage delivery followed by surgery—open repair or TEVAR is—preferred. For complicated type B aortic dissections before 28 weeks, open surgical repair or TEVAR is completed first, with abortion or continuation of pregnancy to be determined by fetal and maternal monitoring [10]. Postpartum dissections are managed by best‐practice guidelines, the same as all nonpregnant individuals.

Acute medical management for aortic dissection involves impulse therapy in normotensive or hypertensive patients and pain control in patients awaiting surgical intervention. This involves reducing the shear stress across the aortic lumen, thereby mitigating further dissection. Rapid‐acting beta‐blockers such as esmolol and labetalol are exemplary agents in attaining a goal of heart rate < 60/min and systolic blood pressure 100–120 mmHg. Nitroprusside can be considered to augment blood pressure control if needed, although the risks of fetal cyanide toxicity are debated. Hydralazine, while used for gestational hypertension, should be avoided in dissections due to reflex tachycardia.

Surgical management of type A aortic dissection focuses on prevention and treatment of complications such as aortic rupture, retrograde extension of dissection into the aortic root, anterograde dissection into undissected segments, and alleviation of malperfusion syndrome. Historically, in patients with acute type A aortic dissection presenting with renal, mesenteric, or lower extremity malperfusion, guidelines recommended proceeding to immediate operative repair of the arch. More recent studies suggest improved outcomes by alleviating malperfusion with endovascular techniques prior to open repair of the aortic arch, as was performed in our patient. A retrospective study of patients with type A aortic dissection complicated by malperfusion treated with endovascular intervention prior to open surgical repair noted a decrease in mortality from aortic rupture (16% to 4%, *p* = 0.05), the risk of dying from organ failure being 6.6 times higher than dying from aortic rupture (hazard ratio = 6.63; 95% CI, 1.5–29; *p* = 0.01) [11]. A recent meta‐analysis suggested that TEVAR is feasible in patients who cannot tolerate open surgery [12]. Surgical resection typically includes the tear site, aneurysmal aorta, and proximal‐most extent of the dissection. Hemiarch repair portends better outcomes than extensive arch replacement but is not always surgically feasible.

One of the feared complications of TEVAR for dissection is spinal cord ischemia (SCI), with a reported incidence of 3.4% [13]. Various risk factors have been associated with SCI, including extensive exclusion of the aorta by endografts, perioperative hypotension, and coverage of the left subclavian artery. The mechanism of SCI is not completely understood but is thought to be related to occlusion of the arteries supplying the spinal cord from endograft, including the artery of Adamkiewicz and spinal cord collateral arteries, which include the left subclavian, intercostal, lumbar, and internal iliac arteries. Thus, guidelines recommend revascularization of the left subclavian artery when coverage is anticipated during elective TEVAR [9]. This naturally is not always a feasible procedure during operative emergencies. Nonetheless, our patient's graft was distal to the proximal edge of the left subclavian artery. Other measures include higher hemoglobin goals, permissive hypothermia, and perioperative steroid and naloxone use. Augmenting spinal cord perfusion pressures via permissive hypertension and cerebrospinal fluid (CSF) drainage has demonstrated a role in the prevention and treatment of SCI [14]. Our patient had a delay in diagnosis, and at that point, the risks and benefits of CSF drainage became unclear.

At discharge, patients should be referred for genetic screening to identify pathogenic variants of syndromic and nonsyndromic HTAD. If identified, patients' clinical management can be informed by the pathogenic gene and can identify high‐risk family members.

Acute type A aortic dissection is life threatening, with an early mortality rate of 1%–2% per hour [6] following symptom onset in nonpregnant individuals. However, patients with pregnancy‐related aortic dissections are younger with fewer comorbid conditions, which tends to portend better survival outcomes. Literature regarding early maternal mortality varies between 6.8% and 31%. Fetal survival prior to 28 weeks is significantly lower compared to after 28 weeks of gestation (14% vs. 94%) [4].

## Author Contributions

**Mohammad Baseem Shaikh:** conceptualization, project administration, validation, writing – original draft. **Reagan Michelle Stafford:** writing – review and editing. **Erinn Ogburn:** supervision, writing – review and editing. **Samuel Tyagi:** writing – review and editing.

## Consent

Written informed consent was obtained from the patient to publish this report in accordance with the journal's patient consent policy.

## Conflicts of Interest

The authors declare no conflicts of interest.

## Data Availability

Data sharing not applicable to this article as no datasets were generated or analysed during the current study.
